# Host FIH-Mediated Asparaginyl Hydroxylation of Translocated *Legionella pneumophila* Effectors

**DOI:** 10.3389/fcimb.2017.00054

**Published:** 2017-03-06

**Authors:** Christopher Price, Michael Merchant, Snake Jones, Ashley Best, Juanita Von Dwingelo, Matthew B. Lawrenz, Nawsad Alam, Ora Schueler-Furman, Yousef A. Kwaik

**Affiliations:** ^1^Department of Microbiology and Immunology, College of Medicine, University of LouisvilleLouisville, KY, USA; ^2^Department of Medicine-Renal, College of Medicine, University of LouisvilleLouisville, KY, USA; ^3^Center for Predictive Medicine, College of Medicine, University of LouisvilleLouisville, KY, USA; ^4^Department of Microbiology and Molecular Genetics, Institute for Medical Research Israel-Canada (IMRIC), Faculty of Medicine, Hadassah Medical School, The Hebrew University of JerusalemJerusalem, Israel

**Keywords:** FIH, asparagine hydroxylation, ankyrin, Dot/Icm, AnkB, hypoxia-inducible factor (HIF), bacterial pathogenesis, *Legionella*

## Abstract

FIH-mediated post-translational modification through asparaginyl hydroxylation of eukaryotic proteins impacts regulation of protein-protein interaction. We have identified the FIH recognition motif in 11 *Legionella pneumophila* translocated effectors, YopM of *Yersinia*, IpaH4.5 of *Shigella* and an ankyrin protein of *Rickettsia*. Mass spectrometry analyses of the AnkB and AnkH effectors of *L. pneumophila* confirm their asparaginyl hydroxylation. Consistent with localization of the AnkB effector to the *Legionella*-containing vacuole (LCV) membrane and its modification by FIH, our data show that FIH and its two interacting proteins, Mint3 and MT1-MMP are acquired by the LCV in a Dot/Icm type IV secretion-dependent manner. Chemical inhibition or RNAi-mediated knockdown of FIH promotes LCV-lysosomes fusion, diminishes decoration of the LCV with polyubiquitinated proteins, and abolishes intra-vacuolar replication of *L. pneumophila*. These data show acquisition of the host FIH by a pathogen-containing vacuole and that asparaginyl-hydroxylation of translocated effectors is indispensable for their function.

## Introduction

Exploitation of host post-translational machineries by translocated bacterial effector proteins is a growing paradigm for effector sub-cellular localization and function in the host cell. Bacterial effectors that are injected into the host cell can be modified by host post-translational modifications including ubiquitination, sumoylation, phosphorylation, and various lipidations including prenylation, palmitoylation, S-acylation, and myristoylation (Kubori and Galan, 2003; Reinicke et al., 2005; Lewis et al., 2008; Patel et al., 2009; Ivanov et al., 2010; Price et al., 2010b; Gaus et al., 2011; Hicks et al., 2011; Hayashi et al., 2013; Beyer et al., 2014). For example, the injected SopE and SptP effectors of *Salmonella* and YopE of *Yersinia* are ubiquitinated by the host resulting in their turnover by proteasome-mediated degradation, while ubiquitination of the SopB effector of *Salmonella* and farnesylation of AnkB of *L. pneumophila* direct sub-cellular localization and impacts function of these injected effectors (Kubori and Galan, 2003; Patel et al., 2009; Ivanov et al., 2010; Price et al., 2010b; Gaus et al., 2011). Therefore, uncovering the host-mediated post-translational modifications of injected bacterial effectors is crucial in understanding function.

Although intracellular bacterial pathogens have been shown to exploit various host post-translational machineries, their exploitation of the host asparaginyl hydroxylation post-translational modification has never been described. The 2-oxoglutarate dioxygenase, designated as factor inhibiting HIF1 (FIH), is a key eukaryotic enzyme, which selectively hydroxylates an asparagine residue within the L(X)5[D/E]φNφ motif (φ represents aliphatic amino acids) in eukaryotic proteins (Hewitson et al., 2002; Lando et al., 2002a,b; Cockman et al., 2009). The addition of the strongly electronegative oxygen atom increases both polarity of a protein and can act as a hydrogen bond donor and acceptor. Therefore, hydroxylation can function as a “molecular switch” for protein-protein interactions (Loenarz and Schofield, 2011). FIH plays a key role in various cellular processes and in particular, it regulates the activity of hypoxia-inducible factor (HIF1), which is the master transcriptional regulator of hypoxia (Webb et al., 2009). During normoxia, HIF1 is hydroxylated by FIH on an asparagine residue and this modification acts as a molecular switch to prevent interaction with its co-activator p300/CBP, blocking transcription of hundreds of HIF1-regulated genes involved in oxygen homeostasis, energy production and immune responses (Hewitson et al., 2002; Lando et al., 2002a,b). In addition, FIH catalyzes asparaginyl hydroxylation of approximately 20 ankyrin repeat domain-containing (ARD) proteins such as p105 and IκBα (Cockman et al., 2009). FIH-dependent hydroxylation of the ARD protein, ASPP2, is required for binding of this protein to its target Par-3 (Janke et al., 2013). Therefore, asparaginyl hydroxylation acts as a molecular switch to promote or reduce protein-protein interactions between HIF1-p300/CBP and ASPP2-Par3 (Hewitson et al., 2002; Lando et al., 2002a,b; Janke et al., 2013). Additionally, FIH hydroxylates the deubiquitinase OTUB, which appears to regulate cellular metabolism (Scholz et al., 2016). A recent study has revealed a complex FIH interactome with many proteins that may serve as substrates for FIH enzyme activity, thus greatly expanding the number of eukaryotic proteins modified by asparaginyl hydroxylation (Rodriguez et al., 2016). However, the biological consequence of asparaginyl hydroxylation of eukaryotic proteins largely remains unclear.

When *L. pneumophila* invades amoebae or human macrophages, it evades the default endosomal-lysosomal degradation pathway and remodels its phagosome into a specialized ER-derived vacuole via intercepting ER-to-golgi vesicular traffic (Isberg et al., 2009; Al-Quadan et al., 2012; Price et al., 2014). This is achieved by the translocation of ~300 effector proteins via the Dot/Icm type IVB secretion system (de Felipe et al., 2008; Isberg et al., 2009; Zhu et al., 2011). These effectors modulate a myriad of eukaryotic processes including host signaling, vesicular trafficking, protein synthesis, apoptosis, prenylation, ubiquitination, and proteasomal degradation (Al-Quadan et al., 2012; Price et al., 2014). Surprisingly, very few of these effectors are essential for intracellular replication of *L. pneumophila*, suggesting specific requirements for different effectors in different environmental hosts.

The AnkB translocated effector is essential for proliferation of *L. pneumophila* within the two evolutionarily-distant hosts, mammalian and protozoan cells, and for intrapulmonary bacterial proliferation and manifestation of pulmonary disease in the mouse model (Al-Khodor et al., 2008; Price et al., 2009, 2010a,b, 2011; Lomma et al., 2010). Recent characterization of the crystal structure of AnkB has confirmed that it is a non-canonical F-box protein with three ankyrins repeats domain (Price et al., 2009; Lomma et al., 2010; Wong et al., 2017). The crystal structure has also confirmed that the F-box domain of AnkB interacts with the host SCF1 ubiquitin ligase, which explains show AnkB functions as a platform for the docking of polyubiquitinated proteins to the *Legionella*-containing vacuolar (LCV) membrane within macrophages and amoebae (Price et al., 2009; Lomma et al., 2010; Wong et al., 2017). The AnkB-assembled polyubiquitinated proteins are predominately Lys48-linked that are ultimately degraded by the host proteasome machinery, which generates higher levels of cellular amino acids that are the main sources of carbon and energy to power replication of *L. pneumophila* (Price et al., 2011). This enables intracellular bacteria to overcome host limitation of essential nutrients and favorable sources of carbon and energy, such as amino acids (Price et al., 2011; Abu Kwaik and Bumann, 2013).

Here we show that 11 *L. pneumophila* type IVB-translocated effectors including, AnkB and AnkH, harbor the recognition motif for FIH-dependent asparaginyl-hydroxylation. Furthermore, the FIH recognition motif is found in translocated effectors from other intracellular microbial pathogens including YopM from *Yersinia pestis*, IpaH4.5 of *Shigella flexneri* and a putative translocated ARD-protein of *Rickettsia felis*. We show that the AnkH and AnkB effectors are modified by asparaginyl hydroxylation. The LCV recruits FIH, which is indispensable for intra-vacuolar proliferation of *L. pneumophila* and plays a partial role in the ability of the LCV to evade lysosomal fusion and is needed for AnkB-dependent assembly of polyubiquitinated proteins on the LCV. This is the first example of an injected microbial effectors post-translationally modified by asparaginyl hydroxylation.

## Materials and methods

### Bacterial strains and cell cultures

*L. pneumophila* strain AA100/130b (ATCC BAA-74), the isogenic mutants, *dotA* and *ankB* or complemented *ankB* mutants were grown as described previously (Al-Khodor et al., 2008). Maintenance of HEK293T cells was performed as previously described (Price et al., 2009). Human monocyte-derived macrophages (hMDMs) were isolated from healthy donors as described previously (Price et al., 2009). Substitutions in the *ankB* gene were performed using standard molecular biology techniques, and the resulting alleles were used to complement the AA100 *ankB* mutant. All methods were carried out and approved in accordance to the University of Louisville Institutional Review Board guidelines and blood donors gave informed consent as required by the University of Louisville Institutional Review Board (IRB # 04.0358).

### qPCR of HIF1-dependent genes

To analyze the effect of the FIH inhibitor, N-oxalyl-D-alanine on FIH enzyme activity, the expression level of four HIF1-dependent genes were chosen as a readout. A total of 3 × 10^6^ hMDMs were seeded into 6-well plates and incubated with and without 8 mM N-oxalyl-D-alanine or 1 mM dimethyloxalylglycine (Enzo) for 8 h. Total RNA was isolated from the hMDMs using a RNeasy Mini Kit (Qiagen) according to the manufacturer's instruction. Purified RNA was converted to first strand cDNA using Superscript III (Invitrogen) and then analyzed by qPCR. Primers specific to GLUT1 (5′-AACTCTTCAGCCAGGGTCCAC-3′, 5′-CACAGTGAAGATGATGAAGAC-3′), GLUT3 (5′-ACTTTGACGGACAAGGGAAATG-3′, 5′- ACCAGTGACAGCCAACAGG-3′), LDHA (5′- ACCCAGTTTCCACCATGATT-3′, 5′- CCCAAAATGCAAGGAACACT-3′) and PGK1 (5′-ATGGATGAGGTGGTGAAAGC-3′, 5′- CAGTGCTCACATGGCTGACT-3′) were analyzed relative to the house-keeping control ACTB (5′- GACAGGATGCAGAAGGAGATCACT-3′, 5′- TGATCCACATCTGCTGGAAGGT-3′). qPCR was performed using PerfeCTa Sybr supermix (Quanta Biosciences) using a StepOne Plus qPCR machine (Applied Biosystems). Determination of fold change in gene expression was calculated using REST-XL software (Pfaffl et al., 2002).

### Co-immunoprecipitation of Skp1 and *ankB* mutants

To assess interaction of the AnkB mutants with the known host target protein, Skp1, HEK293T cells were co-transfected with p3XFLAG CMV 10-AnkB or its mutant alleles and pHA-Skp1. Briefly, a total of 1 × 10^6^ HEK293T cells were seeded into 6-well plates, and the following day transfected with plasmid DNA using polyethyleneimine for 24 h. Following transfection, 3XFLAG AnkB was immunoprecipitated using anti-FLAG M2 magnetic beads (Sigma) according to the manufacturer's instructions. Purified 3XFLAG-AnkB was then subjected to western blot analysis using anti-FLAG M2 antibody (Sigma). Blots were then stripped and reprobed with anti-HA antibody (Santa Cruz), to determine if HA-Skp1 co-immunoprecipitated with AnkB using standard procedures.

### Translocation assay

To assess translocation of the AnkB mutant alleles by *L. pneumophila* during infection of host cells, adenylate cyclase fusions were generated using standard molecular biology techniques. A total of 1 × 10^6^ hMDMs were infected with *L. pneumophila* harboring plasmids expressing various adenylate cyclase fusions at an MOI of 20 for 1 h. Cells were then lysed and processed to assess cAMP concentration by ELISA using the Direct cAMP ELISA kit (Enzo) according to the manufacturer's instructions.

### Localization of FIH, Mint3, MT1-MMP, and polyubiquitin to the LCV

Dependent upon the experiment, the wild type strain, the isogenic mutants *dotA* and *ankB*, and complemented *ankB* mutants were grown on BCYE agar for 3 days at 37°C prior to infection. A total of 5 X 10^5^ hMDMs were seeded into 24-well plates containing sterile glass coverslips and infected with *L. pneumophila* at an MOI of 10 for 1 h, resulting in cells infected with a single bacterium. The hMDMs were treated for 1 h with gentamicin to kill remaining extracellular bacteria and then fixed and permeabilized with −20°C methanol for 5 min. The hMDM monolayers were labeled with rabbit anti-FIH, Mint3, or MT1-MMP antibody (1/200 dilution, Santa Cruz) or mouse FK1 antibody (1/100 dilution, Enzo). *L. pneumophila* were labeled with mouse or rabbit anti-*L. pneumophila* antiserum (1/1,000 dilution) for 1 h. Corresponding Alexa-fluor conjugated secondary antibodies were used for visualization (1/4,000 dilution, Invitrogen). Monolayers were analyzed by confocal microscopy using an Olympus FV1000 scanning fluorescence confocal microscope. On average, 8–15 0.2 um serial Z sections of each image were captured and stored for further analyses, using Adobe Photoshop CS5. For “polyubiquitin cloud” area measurements, Z-stack images of ubiquitin positive LCVs were analyzed using FV10-ASW 3.1 software (Olympus). Co-localization was assessed through visual inspection of Z-stack images using FV10-ASW 3.1 software (Olympus).

### Intracellular replication of *L. pneumophila* in hMDMs

The wild type strain and the isogenic mutant *dotA* were grown on BCYE agar for 3 days at 37°C prior to infection. A total of 1 × 10^5^ hMDMs were plated in 96-wells and treated with and without increasing concentrations of the FIH inhibitor, N-oxalyl-D-alanine (Enzo) for 2 h prior to infection. The hMDM monolayers were infected with *L. pneumophila* at an MOI of 10 for 1 h and then treated for 1 h with gentamicin to kill remaining extracellular bacteria At 2, 24, and 48 h post-infection the hMDMs were lysed with sterile water and *L. pneumophila* CFUs were determined by plating serial dilutions onto BCYE agar. The inhibitor was present throughout the course of the infection and did not affect viability of the hMDMs or *L. pneumophila*. Experiments were performed in triplicate.

### FIH RNAi

HEK293T cells seeded into 24-well plates with glass coverslips were transfected with and without Silencer Select Negative Control #1 or FIH-specific siRNA (sense 5′-Gauaaaagguuacaaacgatt-3′, antisense 5′-Ucguuuguaaccuuuuauctg-3′) (Ambion) using Lipofectamine RNAiMax (Invitrogen) following the manufacturers' instructions for 24 h prior to infection with *L. pneumophila*. To assess knockdown of FIH in transfected cells, cell monolayers were lysed using M-PER reagent (Pierce) and then run on SDS-PAGE gels (BioRad) and analyzed by immunoblotting using a goat anti-FIH antibody (1/200 dilution) (Santa Cruz) and detected using an anti-goat HRP-antibody conjugate (Thermo Scientific). Immunoblots were then immediately stripped and reprobed with an anti-β-tubulin antibody (1/1,000 dilution) (Santa Cruz) to show equal protein loading between lanes.

### Replicative vacuole analysis of *L. pneumophila*

A total of 2 × 10^5^ RNAi treated HEK293T cells as described above or hMDMs were added to 24-well plates containing glass coverslips. The cell monolayers were infected with wild type *L. pneumophila* at an MOI of 20 for 1 h (HEK293T cells) or the wild type, *dotA* or *ankB* mutants, or complemented *ankB* mutants (hMDMs, MOI 10), and then the monolayers were treated with gentamicin for 1 h to kill remaining extracellular bacteria. Following extensive washing to remove gentamicin, the infection proceeded for 10 h. At 10 h the monolayers were permeabilized and fixed using 100% methanol held at −20°C for 5 min, and then labeled with rabbit anti-*Legionella* antiserum (1/1,000 dilution) and counter-labeled with Alexa-Fluor 488 anti-rabbit antibody (1/4,000 dilution, Invitrogen) and DAPI to stain the nuclei. Monolayers were examined by confocal microscopy. A total of 100 replicative vacuoles from 100 individual cells were analyzed for each experimental condition and performed in triplicate.

### Vesicular trafficking of *L. pneumophila* in hMDMs

The wild type strain was grown on BCYE agar for 3 days at 37°C. For killed bacteria, bacterial cells were resuspended in PBS with 3.7% formalin and incubated for 30 min at room temperature and then washed 3 times with PBS to remove residual formalin. A total of 5 × 10^5^ hMDMs were seeded into 24-well plates containing sterile glass coverslips and treated with and without increasing concentrations of the FIH inhibitor, N-oxalyl-D-alanine (Enzo) for 2 h prior to infection. The hMDM monolayers were infected with live and formalin killed *L. pneumophila* at an MOI of 10 for 1 h. A total of 2 × 10^5^ HEK293T cells transfected with control or FIH RNAi were seeded into 24-well plates containing sterile glass coverslips were infected with wild type bacteria at an MOI of 20 for 1 h. For both hMDMs and HEK293T cells, the monolayers were treated for 1 h with gentamicin to kill remaining extracellular bacteria and then the cells were fixed and permeabilized with −20°C methanol for 5 min. The cell monolayers were labeled with rabbit anti-*L. pneumophila* antiserum (1/1,000 dilution) and mouse anti-LAMP2, CathD, or KDEL (1/2,000, 1/100, and 1/200 dilutions respectively, Transduction Labs, Stressgen). Anti-mouse IgG Alexa-fluor 555 and anti-rabbit IgG Alexa-fluor 488 secondary antibodies (1/4,000 dilution, Invitrogen) were used to visualize vacuolar markers *L. pneumophila* respectively. A total of 100 LCVs from 100 individual cells were analyzed by confocal microscopy for each experimental condition and performed in triplicate.

### Mass spectrometry

The analysis for the presence and extent of asparagine (N) hydroxylation was conducted as described by Petkowski et al. (2013) with some modifications (Petkowski et al., 2013) as described below. A colloidal Coomassie blue stained, 1D-SDS-PAGE gel band was excised, de-stained and equilibrated into 0.1 M triethylammonium bicarbonate, pH 8.5 and digested with 80 ng mass spectrometry grade trypsin (Promega, Madison, WI) per gel plug. The digest supernatant was transferred to a new tube and the gel plugs extracted using a modification of Shevchenko et al. (1996). A 5 ul aliquot of this peptide solution was loaded onto a Dionex Acclaim PepMap 100 75 μm × 2 cm, nanoViper (C18, 3 μm, 100Å) trap, and resolved on a Dionex Acclaim PepMap RSLC 50 μm × 15 cm, nanoViper (C18, 2 μm, 100Å). The sample was eluted using a linear 2 to 60% acetonitrile gradient using an EASY nano1000 UHPLC and introduced into a LTQ-Orbitrap ELITE mass spectrometer (ThermoElectron, Waltham, MA) for accurate mass measurements using a Nanospray Flex Ion Source (ThermoElectron, Waltham, MA), a stainless steel emitter with a capillary temperature set to 225 C and a spray voltage of 1.6 kV and lock mass enabled (0% lock mass abundance) for the 371.101236 m/z polysiloxane peak as an internal calibrant (Cox et al., 2011). Data dependent tandem mass spectra were collected using HCD and ETD fragmentation using an Nth Order Double Play with ETD Decision Tree method (Swaney et al., 2008) was created in Xcalibur v2.2 and included a parent mass list for +2 or +3 charge states of tryptic peptides (0, 1, or 2 miss cleavages) based on the protein sequence and putative hydroxylation. Targeted and data dependent spectra were acquired and searched using MASCOT ver 2.1 and Sequest through Proteome Discoverer 1.4 using the 2/13/2013 version of the UniprotKB *Homo sapiens* reference proteome (canonical and isoform sequences) appended with the *Legionella* sp proteins considering up to two missed cleavages, and N-hydroxylation as variable modifications. The false discovery rate was controlled by use of (1) a decoy database generated from this database with the program decoy.pl (matrixscience.com) and use of Peptide & Protein Prophet algorithm to report FDRs below 1%.

## Results

### Identification of the FIH consensus motif in translocated effectors of *L. pneumophila* and other bacterial pathogens

To date only HIF1 and ~20 human ARD proteins are known to be hydroxylated by FIH (Hewitson et al., 2002; Lando et al., 2002a,b; Cockman et al., 2009). *L. pneumophila* translocates at least 11 ARD proteins by its Dot/Icm type IVB translocation system into the host cell upon invasion, including those that contribute to intracellular replication (AnkB, H, J) (Al-Khodor et al., 2008; Habyarimana et al., 2008; Pan et al., 2008; Price et al., 2011). Therefore, we examined the ARD proteins of *L. pneumophila* for the presence of the FIH consensus motif. The AnkB, AnkH, and AnkN effectors all harbor the FIH consensus motif in the ARD regions, suggesting that these effectors are potential targets for FIH asparaginyl hydroxylation upon their translocation into the host cell (Table S1). *In silico* analysis of the whole *L. pneumophila* genome identified 8 additional effector proteins that harbor the FIH recognition motif (Table S1).

**Figure 1:**
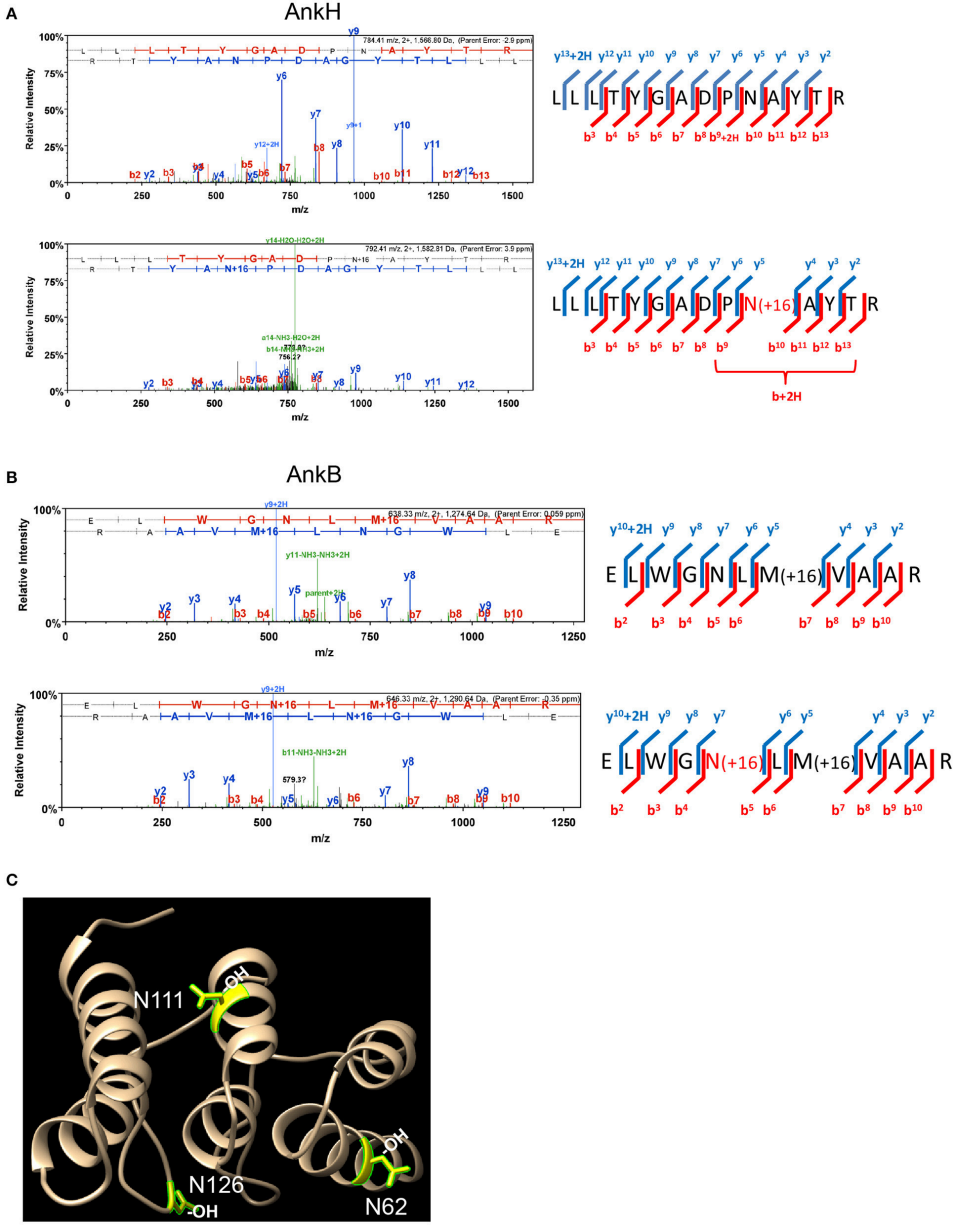
**AnkH and AnkB are modified by host asparaginyl hydroxylation**. High resolution LCMS analysis 1D-LC-LTQ-Orbitrap-ELITE-MS of AnkH and AnkB protein expressed in HEK293T cells identifies hydroxylated asparagine residues. **(A)** AnkH: (Top) HCD fragmentation spectrum for +2 charged ion with a monoisotopic m/z: 1,566.81 Da; (Bottom) HCD fragmentation spectrum for +2 charged ion with a monoisotopic m/z: 1,582.81 Da; ProteomeDiscover v1.3 analysis of upper MS/MS data set identifies a tryptic peptide (Mascot Ion Score 77.8, Sequest Xcorr 3.94, and deltaCn 0.49) with the sequence LLLTYGADPNATYTR. Analysis of lower MS/MS data set identifies a tryptic peptide (Mascot Ion Score 19.7, Sequest Xcorr 2.33, and deltaCn 0.30) with the sequence LLLTYGADPN(OH)ATYTR. Both peptides were observed with sub-5 ppm mass accuracy and with near complete b-ion (red hash; includes b-, b^*^-, and b+2H+- ions) and y-ion (blue hash; includes y-. y^*^-, and y+2H+-) coverage of parent ions. **(B)** AnkB: (Top) CID fragmentation spectrum for +2 charged ion with a monoisotopic m/z: 1,274.64 Da; (Bottom) CID fragmentation spectrum for +2 charged ion with a monoisotopic m/z: 1,290.64 Da; ProteomeDiscover v1.4 analysis of upper MS/MS data set identifies a tryptic peptide (Mascot Ion Score 81.7) with the sequence ELWGNLMVAAR. Analysis of lower MS/MS data set identifies a tryptic peptide (Mascot Ion Score 41.1) with the sequence ELWGN(OH)LMVAAR. Both peptides were observed with <1 ppm mass accuracy and with near complete b-ion (red hash; includes b- ions) and y-ion (blue hash; includes y-. y^*^-, and y+2H+-) coverage of parent ions. **(C)** X-ray crystal structure of the AnkB ARD region illustrating the hydroxylated asparagine residues shown in yellow.

To assess whether some of the 11 *L. pneumophila* translocated effectors and YopM from *Y. pestis* that harbor the FIH consensus sequence are hydroxylated, LC-MS MS1 analysis was performed. Purification of translocated effector proteins endogenously produced by intracellular bacteria during infection of host cells results in insufficient material for analysis. To overcome this limitation, the effector proteins were expressed and purified from transiently transfected HEK293T cells. Of the twelve translocated effectors, only AnkN, SdeC, SdcA, LepB, AnkH, AnkB, and YopM could be reliably expressed in HEK293T cells and purified in sufficient quantities for mass spectrometry analysis of post-translational modification. LC-MS MS1 analysis of AnkH protein purified from HEK293T cells assigned a site of hydroxylation on the 92N residue within the predicted FIH recognition motif in the second ankyrin repeat of AnkH (Table S1, Figure 1A). LC-MS MS1 analysis of AnkB revealed three hydroxylation sites, on residues 62N, 111N, and 126N (Figure 1B, Figure S1), while detection of hydroxylation sites within YopM was not reproducible in three attempts. Although the hydroxylated asparagine residues in AnkB were not part of the predicted FIH consensus motif, they were within the three-ankyrin repeat containing domain (Wong et al., in press) (Table S1, Figures 1B,C and Figure S1). LC-MS MS1 analyses of the AnkN, SdeC, SdcA, LepB effectors did not reveal asparaginyl hydroxylation modification. Therefore, our data shows that at least 2 out of the 7 effectors analyzed are modified by asparaginyl hydroxylation.

### Acquisition of FIH, Mint3, and MT1-MMP by the LCV

**Figure 2:**
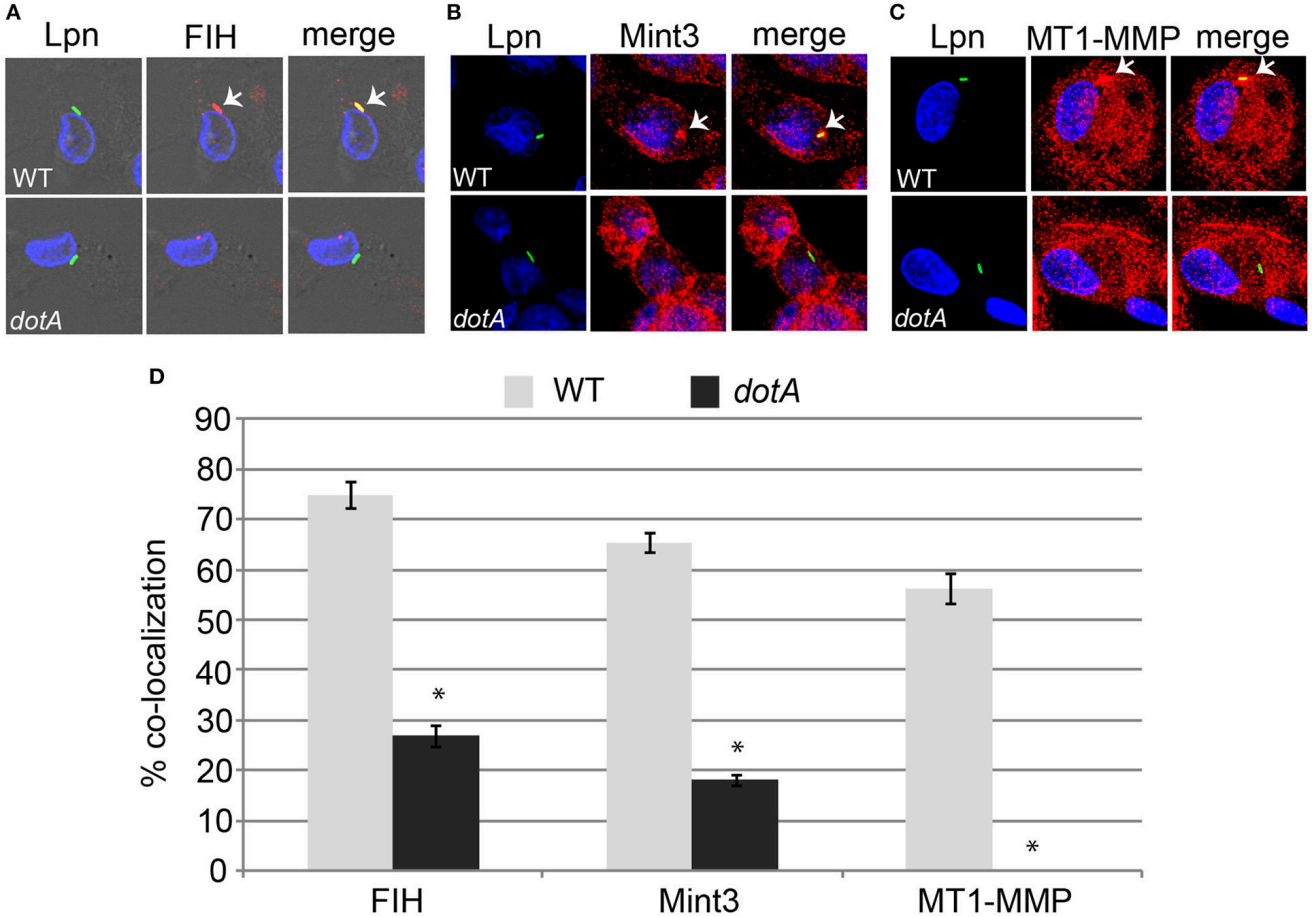
**FIH, Mint3, and MT1-MMP are recruited to the LCV in a Dot/Icm-dependent manner**. hMDMs were infected with wild type *L. pneumophila* or the isogenic *dotA* mutant for 2 h to determine co-localization of the LCV with FIH **(A)**, Mint3 **(B)**, and MT1-MMP **(C)**. Bacteria were labeled with anti-Lpn antiserum (green) and FIH, Mint3, or MT1-MMP was labeled with specific antibodies (red) and then analyzed by confocal microscopy. The arrows indicate co-localization of FIH, Mint3, or MT1-MMP with the LCV. Quantification of % co-localization is shown in **(D)** and data represents (mean ± SD, *n* = 100 LCVs) of the frequency of acquisition of FIH, Mint3, or MT1-MMP by the LCV. ^*^above the *dotA* bars indicates statistically significant difference in % co-localization compared to the corresponding wild type LCVs (unpaired *t*-test, *p* < 0.01). The data is representative of three independent experiments.

The AnkB effector is localized to the LCV membrane through host-mediated farnesylation (Price et al., 2010b). In addition, the effectors LepB, SdeC, and SdcA which are potential candidates for FIH-mediated asparaginyl hydroxylation are also LCV-localized (Chen et al., 2004, 2007; Luo and Isberg, 2004; Bardill et al., 2005; Ingmundson et al., 2007; Tan et al., 2011). Since FIH is a cytosolic enzyme that can be sequestered to membranous structures such as the Golgi apparatus through interaction with Mint3 and MT1-MMP in macrophages (Sakamoto and Seiki, 2009, 2010) and the LCV intercepts ER-Golgi vesicular traffic (Isberg et al., 2009; Al-Quadan et al., 2012; Price et al., 2014), we determined if FIH and its two interacting partners (Mint3 and MT1-MMP) were recruited to the LCV within hMDMs. The data showed that by 2 h of infection, 75, 65, and 56% of the LCVs harboring wild type bacteria co-localized with FIH, Mint3, and MT1-MMP, respectively (Figures 2A–D). In contrast, only 27, 18.2, and 0% of the LCVs harboring the *dotA* translocation-deficient mutant co-localized with FIH, Mint3, and MT1-MMP, respectively (Figures 2A–D) and this was significantly reduced relative to co-localization observed for wild type LCVs (unpaired *t*-test, *p* < 0.01). This indicates recruitment of these proteins to the LCV is dependent on the Dot/Icm T4SS apparatus.

### Role of 62N, 111N, and 126N on *ankB* function

**Figure 3:**
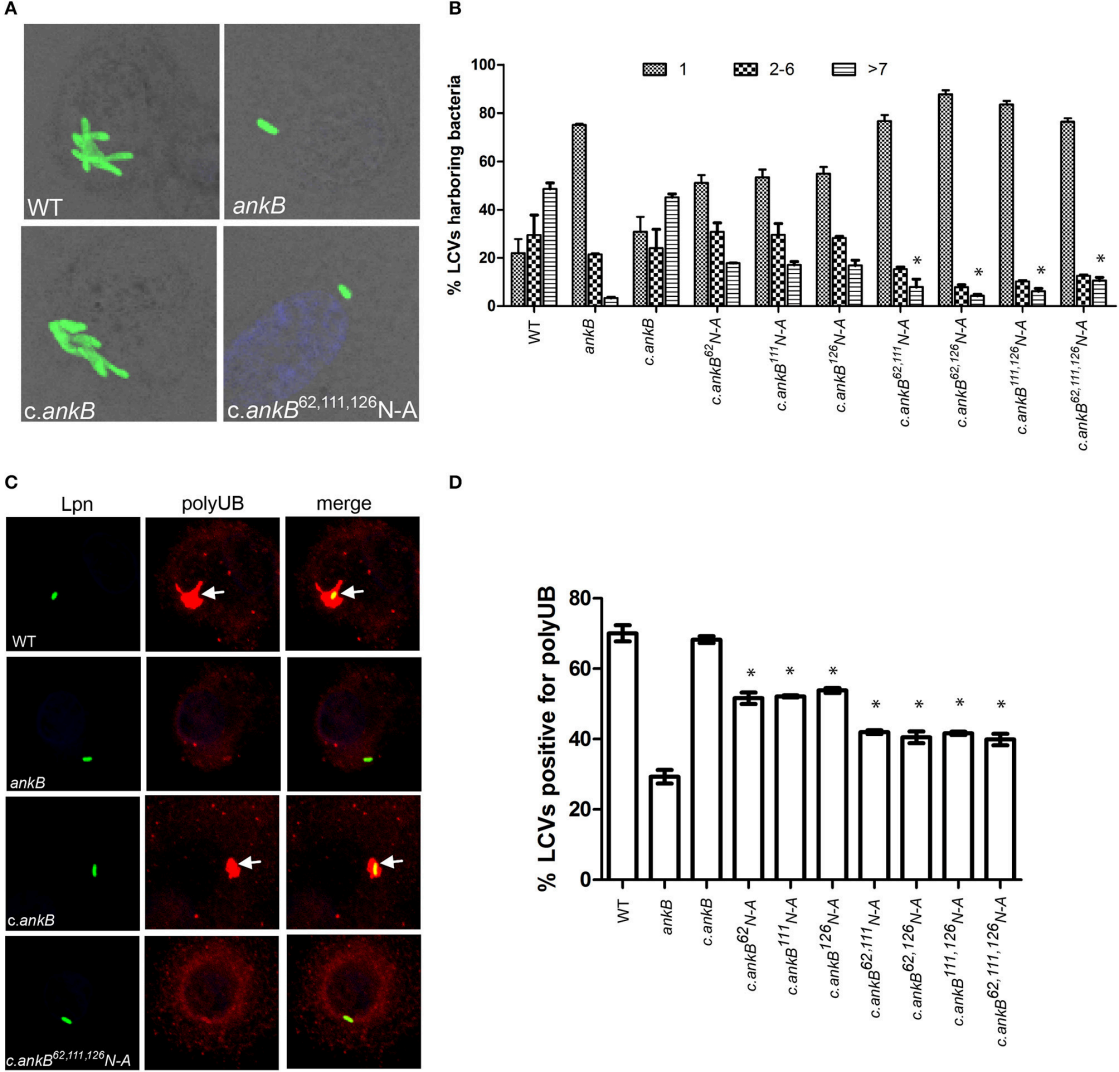
**Amino acid residues 62N, 111N, and 126N are required for AnkB function. (A)** To determine intra-vacuolar replication at 10 h post-infection, hMDMs were infected with either wild type *L. pneumophila* (WT), *ankB* mutant or complemented *ankB* mutants (c.*ankB*) and then labeled with anti-Lpn antiserum (green). Images are representative of over 100 infected cells. **(B)** Quantitation of replicative vacuole analysis in hMDMs. The numbers of bacteria were determined by analyzing Z-stack confocal images of infected cells, bars represent (mean ± SD, *n* = 100 LCVs) the % total of bacteria per LCV. ^*^above the >7 bacteria bars indicates statistically significant decrease in bacterial numbers relative to that observed for wild type (unpaired *t*-test, *p* < 0.01). **(C)** Representative confocal microscopy images of *L. pneumophila* infected hMDMs at 2 h post-infection to determine % co-localization of polyubiquitinated protein with the LCV. **(D)** Quantitation of the number of LCVs decorated by polyubiquitinated proteins in hMDMs at 2 h post-infection. Bars represent (mean ± SD, *n* = 200 LCVs) and are representative of three independent experiments ^*^above the bars indicates statistically significant decrease in polyubiquitin recruitment relative to that observed for wild type (unpaired *t*-test, *p* < 0.01).

The AnkB F-box effector is required for recruitment of polyubiquitinated proteins to the LCV and intra-vacuolar replication of *L. pneumophila* (Al-Khodor et al., 2008; Price et al., 2009, 2011; Lomma et al., 2010). To determine the functional importance of the three hydroxylated asparagine residues identified in AnkB, the *ankB* mutant was complemented with *ankB* alleles in which each of the three asparagine residues were substituted with alanine and the effect on intra-vacuolar replication and polyubiquitinated protein recruitment to the LCV were determined. Intra-vacuolar growth in human monocyte-derived macrophages (hMDMs) was assessed by enumerating replicative vacuoles at 10 h post-infection using confocal microscopy. The data showed that expression of *ankB* alleles with a single alanine substitution at 62N, 111N, or 126N resulted in a partial defect in intra-vacuolar replication compared to the wild type allele (Figures 3A,B). Strikingly, expression of *ankB* alleles with multiple substitutions significantly reduced the number of LCVs harboring > 7 bacteria compared to wild type LCVs (unpaired *t*-test, *p* < 0.01), approaching that observed for the *ankB* null mutant (Figures 3A,B).

Since AnkB is intimately involved in recruitment of polyubiquitinated proteins to the LCV we next determined the impact of alanine substitutions at 62N, 111N, or 126N on this process. Following 2 h infection of hMDMs, ~70% of LCVs harboring wild type bacteria were decorated with polyubiquitinated proteins compared to only 29% for LCVs harboring the *ankB* mutant (Figures 3C,D). Complementation of the *ankB* mutant with the wild type *ankB* gene restored decoration of the LCV with polyubiquitinated proteins to wild type levels (~70%) (Figures 3C,D). In contrast, decoration of LCVs harboring the *ankB* mutant complemented with *ankB* 62N, 111N, or 126N substitution alleles was significantly reduced to ~50% (unpaired *t*-test, *p* < 0.01) compared to wild type LCVs. Furthermore, when multiple substitutions were made, decoration of the LCV with polyubiquitinated proteins was further reduced to ~40% (unpaired *t*-test, *p* < 0.01) compared to wild type, approaching that observed for the *ankB* mutant (Figure 3C,D). Importantly, substitution of these N residues did not impact translocation of the altered AnkB effectors into host cells compared to the wild type protein and did not impact interaction of this effector with its Skp1 component of the E3 ubiquitin ligase (Figures S2A,B). Taken together, this indicates that these three asparagine residues are important for AnkB function.

### Role for FIH in recruitment polyubiquitinated proteins to the LCV

**Figure 4:**
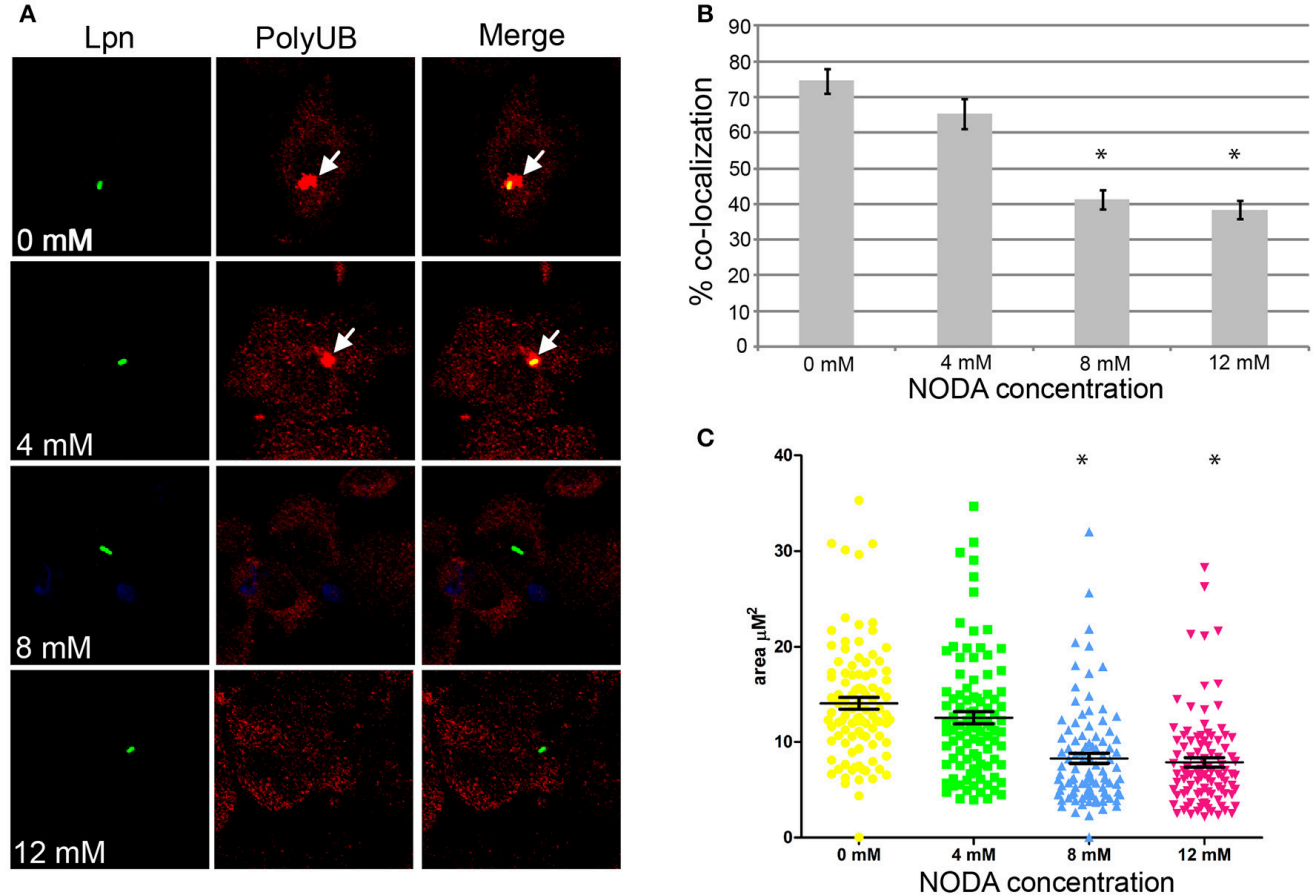
**Docking of polyubiquitinated proteins to the LCV in hMDMs requires FIH activity. (A)** hMDMs pretreated with the FIH inhibitor, NODA (0–12 mM), were infected with wild type *L. pneumophila* for 2 h. Representative confocal microscopy images of infected hMDMs show co-localization of polyubiquitinated protein with the LCV. Quantification of % co-localization is shown in **(B)** and data represents (mean ± SD, *n* = 100 LCVs) of the frequency of acquisition of polyubiquitinated proteins by the LCV, ^*^above the bars indicates statistically significant decrease in polyubiquitin recruitment relative to that observed for untreated cells (unpaired *t*-test, *p* < 0.01). **(C)** Semi-quantitative analysis of the polyubiquitin “cloud” on the LCVs in infected hMDMs treated with increasing concentrations of the FIH inhibitor, 2 h post-infection. Data represents the distribution of polyubiquitin recruitment size (mean ± SD, *n* = 100 LCVs) and is representative of three independent experiments. ^*^above the data points indicates statistically significant decrease in polyubiquitin “cloud”size relative to that observed for untreated cells (unpaired *t*-test, *p* < 0.01).

Assembly of polyubiquitinated proteins at the LCV (Dorer et al., 2006) is mediated by the AnkB effector (Price et al., 2009, 2011; Lomma et al., 2010). Since AnkB is hydroxylated on three asparagine residues and substitution of these residues reduces polyubiquitinated protein recruitment to the LCV, we determined whether FIH activity is required for the assembly of polyubiquitinated proteins by AnkB on the LCV. FIH was chemically inhibited by N-oxalyl-D-alanine (NODA) and the effect on recruitment of polyubiquitinated proteins to the LCV was analyzed. NODA exhibited FIH inhibitory activity in hMDMs, as shown by elevated expression of HIF1-dependent genes by qPCR (Table S2). Following 2 h infection of untreated hMDMs, 75% of the wild type-containing LCVs were decorated with polyubiquitinated proteins (Figures 4A,B). Compared to untreated cells, blocking FIH activity with NODA, caused a dose-dependent inhibition of recruitment of polyubiquitinated proteins with only 38% of the LCVs decorated when 12 mM NODA was used (Figures 4A,B) (unpaired *t*-test, *p* < 0.01 compared to untreated cells), mimicking the effect of substituting the three hydroxylated asparagine residues of AnkB (Figures 3C,D). In addition to the reduced number of LCVs positive for polyubiquitinated protein in hMDMs treated with NODA, microscopic examination indicated that the area of the polyubiquitin “cloud” surrounding the LCV that were positive was reduced. To semi-quantitate the polyubiquitin cloud, the area of the cloud was measured using Z-stack confocal images. The average polyubiquitinated cloud surrounding LCVs in untreated hMDMs was 14.5 μm^2^ while those in hMDMs treated with 12 mM NODA was significantly reduced to only 7 μm^2^ (unpaired *t*-test, *p* < 0.01 compared to untreated cells) with many LCVs showing little extension of the “cloud” away from the periphery of the LCV (Figure 4C, Figure S3). Taken together, these data clearly show that inhibition of FIH had a negative impact on the biological function of AnkB in assembly of polyubiquitinated proteins at the LCV.

### FIH is required for intracellular replication of *L. pneumophila*

**Figure 5:**
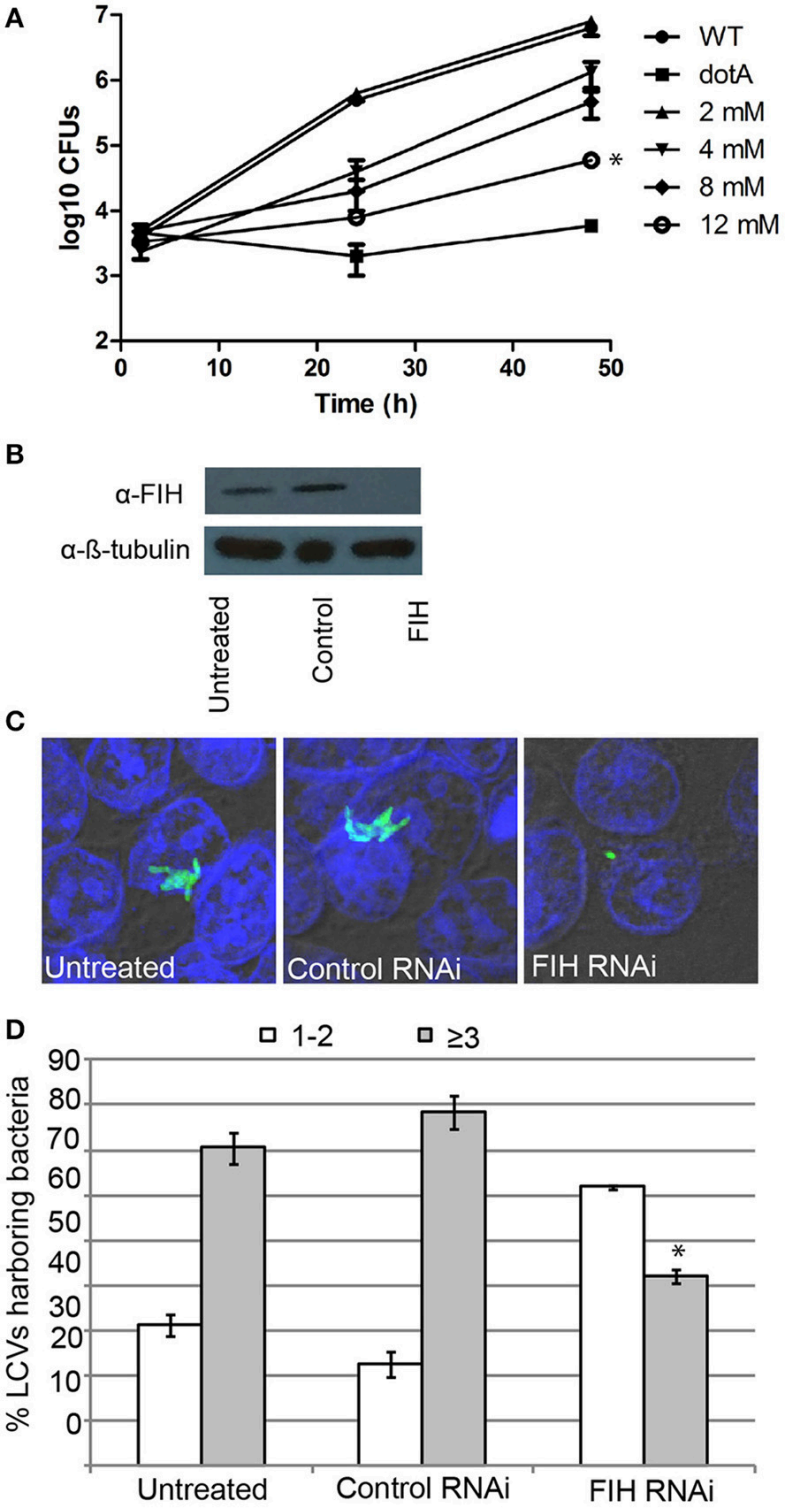
**FIH is indispensable for intracellular proliferation of *L. pneumophila*. (A)** Growth kinetics of *L. pneumophila* in hMDMs treated with increasing concentrations of the FIH inhibitor, (NODA). The hMDMS were infected with the wild type strain or the isogenic *dotA* mutant and then CFUs were determined at 2, 24, and 48 h post-infection. ^*^indicates statistically significant reduced CFUs at 48 h post-infection relative to that observed for wild type (unpaired *t*-test, *p* < 0.01). Data represents (mean ± *SD, n* = 3 infected hMDM monolayers). **(B)** Immunoblots showing specific knockdown of FIH protein expression in HEK293T cells transfected with control or FIH specific RNAi following 24 h. Cell lysates were run on SDS-PAGE, and FIH proteins were detected on a western blot probed with an anti-FIH antibody. The immunoblot was stripped and reprobed using an anti-β-tubulin antibody to show equivalent protein loading between lanes. **(C)** Representative confocal images of *L. pneumophila* replicative vacuoles in untreated HEK293T cells or cells treated with control or FIH specific RNAi at 10 h post-infection. Bacteria are labeled with anti-Lpn anti-serum (green), while the cell nucleus is stained with DAPI (blue). Images are representative of over 100 infected cells. **(D)** Replicative vacuole analysis quantitation in HEK293T cells treated with FIH RNAi. The numbers of bacteria were determined by analyzing Z-stack confocal images of infected cells, bars represent (mean ± SD, *n* = 100 LCVs) the % total of bacteria per LCV. ^*^above the ≥3 bacteria bars indicates statistically significant decrease bacterial numbers relative to that observed for wild type (unpaired *t*-test, *p* < 0.01 and is representative of three independent experiments.

Since FIH is recruited to the LCV in a Dot/Icm-dependent manner, we determined the biological relevance of host FIH on intra-vacuolar replication of *L. pneumophila*. FIH was chemically inhibited by NODA and the effect on intra-vacuolar replication in hMDMs was analyzed. The data showed that NODA inhibited intra-vacuolar replication of *L. pneumophila* in a dose-dependent manner. At 2 mM, NODA had a minor negative effect but at 12 mM, it essentially blocked intracellular replication of *L. pneumophila* compared to untreated cells (Figure 5A). *L. pneumophila* numbers increased by 3.2 log units over 48 h of infection, but in cells treated with 12 mM NODA, only a 1.3 log unit increase was observed and this difference was statistically significant (unpaired *t*-test, *p* < 0.01) (Figure 5A). The addition of 12 mM NODA did not affect viability of the hMDMs or *L. pneumophila* during the course of the experiment and did not affect *L. pneumophila in vitro* growth in broth culture (data not shown). To confirm the effect of chemical inhibition of FIH on intracellular replication, expression of FIH was knocked down in HEK293T cells using specific RNAi (Figure 5B). Intracellular growth was assessed by enumerating replicative vacuoles at 10 h post-infection using confocal microscopy (Figures 5C,D). The data showed that replication of *L. pneumophila* was significantly reduced in HEK293T cells treated with FIH specific RNAi, with only 38% of LCVs harboring replicative LCVs (three or more bacteria/LCV), compared to 69 and 77% of LCVs in untreated or control RNAi-treated cells, respectively (unpaired *t*-test, *p* < 0.01 compared to untreated cells) (Figure 5D). Knockdown of FIH in HEK293T cells did not affect cellular viability during the course of the experiments (data not shown). Taken together, the data clearly show that the function of FIH is indispensable for intra-vacuolar replication of *L. pneumophila*.

### The role of FIH in biogenesis of the LCV

**Figure 6:**
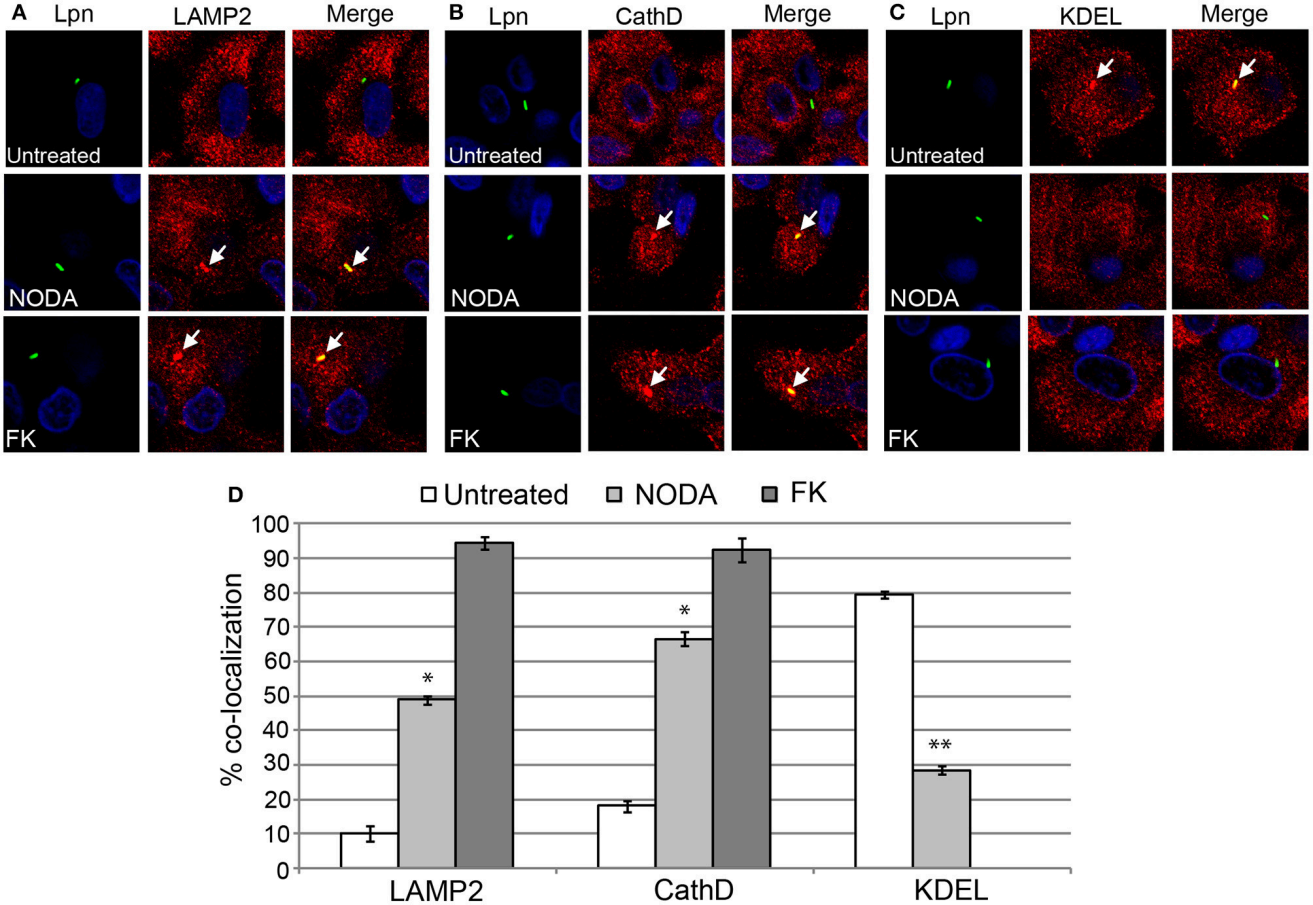
**FIH activity is required for biogenesis of the LCV**. hMDMs untreated or pretreated with the FIH inhibitor, NODA, were infected with live or formalin-killed (FK) wild type *L. pneumophila* for 2 h. Representative confocal microscopy images of infected cells are shown indicating % co-localization of the LCV in hMDMS or HEK293T cells with **(A)** LAMP2, **(B)** CathD, and **(C)** KDEL. Bacteria were labeled with anti-Lpn antiserum (green) and LAMP2, CathD, and KDEL was labeled with specific antibodies (red) and analyzed by confocal microscopy. The arrows indicate co-localization of LAMP2, CathD, and KDEL with the LCV. **(D)** Quantification of the frequency of co-localization of LAMP2, CathD or KDEL with LCVs. Data represents (mean ± SD, *n* = 100 LCVs). ^*^represents statistically significant increase in NODA-treated cells vs. untreated cells (unpaired *t*-test, *p* < 0.01). ^**^represents statistically significant decrease in NODA-treated cells vs. untreated cells (unpaired *t*-test, *p* < 0.01). The data is representative of three independent experiments.

Since blocking FIH had a dose-dependent negative impact on intracellular replication of *L. pneumophila*, we determined whether this growth defect was associated with alterations in the biogenesis of the LCV that is rough ER-derived, and evades lysosomal fusion (Isberg et al., 2009; Al-Quadan et al., 2012; Price et al., 2014). The infected hMDMs were fixed at 2 h post-infection and labeled with antibodies specific for the late endosomal/lysosomal marker, LAMP2, and lysosomal enzyme, cathepsin D, and the ER marker, KDEL. In untreated hMDMs, only 10 and 18% of LCVs harboring live wild type bacteria co-localized with LAMP2 and cathepsin D respectively (Figures 6A,B,D). In contrast, 49 and 66% of LCVs harboring live wild type bacteria in 12 mM NODA-treated hMDMs co-localized with LAMP2 and cathepsin D, and this increase was significant (unpaired *t*-test, *p* < 0.01 compared to untreated cells) (Figures 6A,B,D). As expected, over 90% of LCVs containing formalin-killed bacteria co-localized with LAMP2 and cathepsin D (Figures 6A,B,D). Furthermore, only 28% of LCVs co-localized with the KDEL ER marker in NODA-treated cells compared to 80% in untreated cells (unpaired *t*-test, *p* < 0.01 compared to untreated cells) (Figures 6C,D). No LCVs harboring formalin killed bacteria were positive for KDEL co-localization (Figures 6C,D).

**Figure 7:**
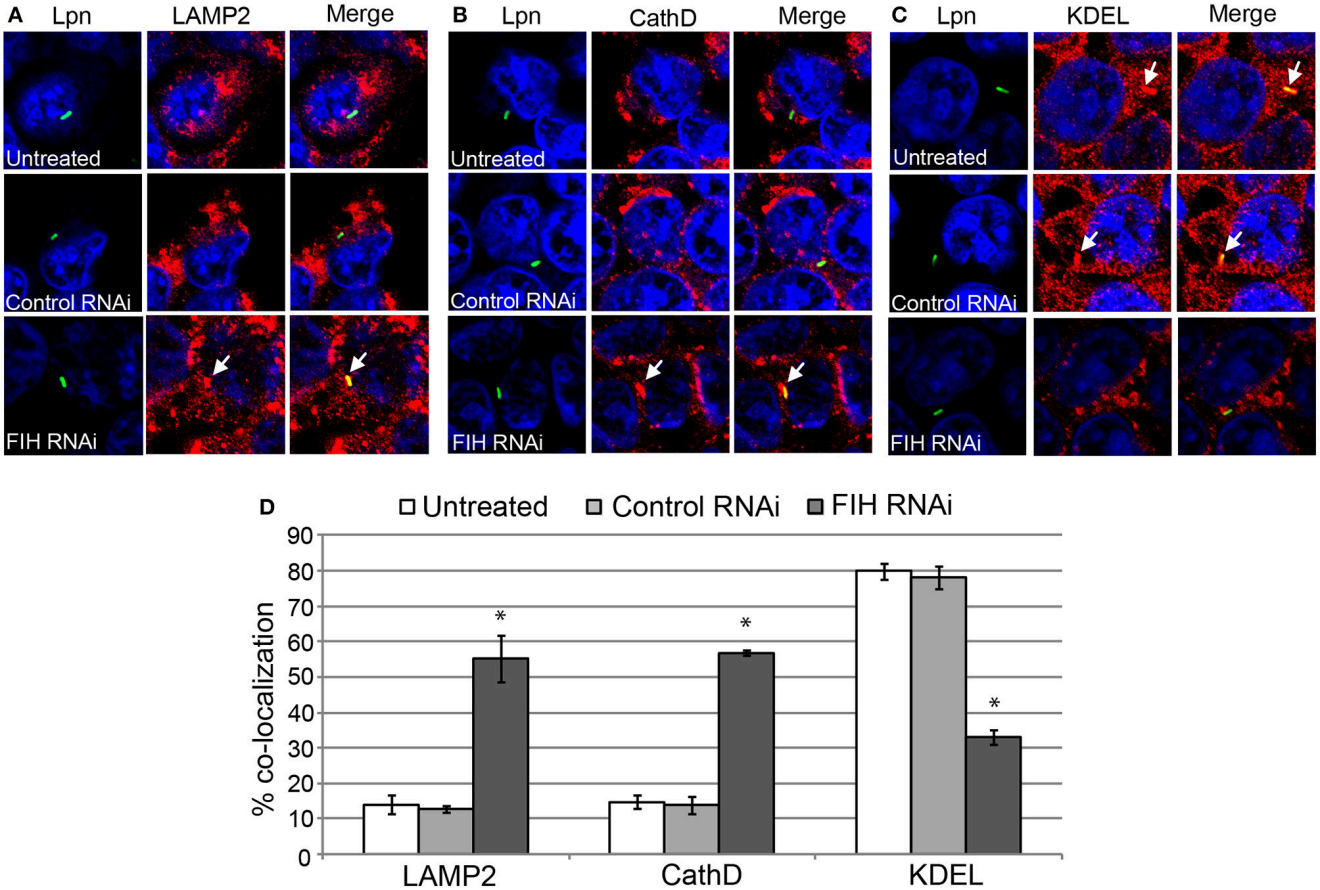
**FIH protein is required for biogenesis of the LCV**. HEK293T cells were transfected with control or FIH-specific RNAi and infected with wild type *L. pneumophila* for 2 h. Representative confocal microscopy images of infected cells are shown indicating % co-localization of the LCV in hMDMS or HEK293T cells with **(A)** LAMP2, **(B)** CathD, and **(C)** KDEL. Bacteria were labeled with anti-Lpn antiserum (green) and LAMP2, CathD, and KDEL was labeled with specific antibodies (red) and analyzed by confocal microscopy. The arrows indicate co-localization of LAMP2, CathD, and KDEL with the LCV. **(D)** Quantification of the frequency of co-localization of LAMP2, CathD, or KDEL with LCVs. Data represents (mean ± SD, *n* = 100 LCVs) ^*^represents statistical significance in FIH RNAi-treated cells vs. untreated or control RNAi-treated cells (unpaired *t*-test, *p* < 0.01). The data is representative of three independent experiments.

To confirm the effect of chemical inhibition of FIH-dependent evasion of lysosomal fusion and remodeling of the LCV by the ER, expression of FIH was silenced using specific RNAi. Similar to NODA, knockdown of FIH significantly increased the number of LCVs co-localized with LAMP2 and cathepsin D, 55 and 57% respectively, compared to untreated or control RNAi treated cells (unpaired *t*-test, *p* < 0.01) (Figures 7A,B,D). In addition, only 33% of LCVs co-localized with the KDEL ER marker compared to 80 and 78% in untreated and control RNAi HEK293T cells, respectively (unpaired *t*-test, *p* < 0.01) (Figures 7C,D). Confirmation of specific and complete FIH knockdown is shown in Figure 5B, as assessment of LCV trafficking and impact on intra-vacuolar replication of *L. pneumophila* was performed in parallel. Taken together, these data clearly show that FIH is partially required for Dot/Icm-dependent lysosomal evasion and ER-mediated remodeling of the LCV.

## Discussion

An emerging theme clearly shows that not only do translocated effectors of bacterial pathogens mimic eukaryotic protein functions, but they are also modified by various eukaryotic post-translational modification machineries (Ivanov and Roy, 2013; Kim et al., 2014). Our data show for the first time that injected effectors are modified by host FIH-dependent asparaginyl hydroxylation.

Our search for bacterial effectors that are potentially modified by host-asparaginyl hydroxylation was based upon the canonical FIH hydroxylation motif that was defined by using sequence data derived from the HIF1 and ARD hydroxylation sites (Hewitson et al., 2002; Lando et al., 2002a,b; Cockman et al., 2009). For AnkH, the motif was identified within the first ankyrin repeat and the predicted asparagine residue was modified by hydroxylation. Until recently, the biological consequence of asparaginyl hydroxylation within ARDs was unclear, however studies have now shown this modification regulates protein-protein interaction of ARD containing proteins, which subsequently affects their functions (Janke et al., 2013; Peng et al., 2014). Though AnkH contributes to intra-vacuolar replication of *L. pneumophila*, its functional role is unknown (Habyarimana et al., 2008, 2010). Further studies will be needed to determine the impact of asparaginyl hydroxylation on the ability of AnkH to mediate potential protein-protein interactions with its substrate, which may in turn affect its function. We also identified a single FIH hydroxylation motif within the ARD region of AnkB, but in contrast to AnkH, the predicted asparagine was not modified. However, we identified three additional asparaginyl hydroxylation sites on AnkB that occurred at distinct sites identified by the motif defined by sequence analysis. Nevertheless, they are located within the three-ankyrin repeat containing domain of AnkB (Wong et al., 2017). Interestingly, one of the hydroxylated residues (N126) occurs in a loop structure adjacent to a putative substrate interacting residue (Y127) (Wong et al., 2017) and therefore, we predict that alteration in local electronegative charge may enhance or interfere with substrate binding. FIH has not been shown to hydroxylate asparagine residues outside of the predicted motif (Wilkins et al., 2012) and therefore it will be interesting to determine if substrate specificity of FIH is wider than expected or that other host enzymes contribute to this post-translational modification. AnkB plays a central role for *L. pneumophila* by promoting the degradation of polyubiquitinated proteins which allows this organism to access essential amino acids that are used for both energy and a carbon source (Price et al., 2009, 2011). Substitution of the three hydroxylated asparagine residues significantly impacts the ability of AnkB to recruit polyubiquitinated proteins to the LCV and concomitantly fails to restore intra-vacuolar replication of an *ankB* mutant strain of *L. pneumophila*. Furthermore, blocking host FIH activity results in a similar phenotype to the AnkB substitutions, and taken together suggests that asparaginyl hydroxylation of AnkB contributes to the function of this effector.

Blocking FIH activity results in a dose-dependent inhibition of intra-vacuolar replication of *L. pneumophila*. Interestingly however, only ~55% of LCVs trafficked to a lysosomal compartment, indicating that the FIH-mediated block in intra-vacuolar replication of *L. pneumophila* has both lysosomal evasion-independent and -dependent mechanisms. Both AnkB and AnkH are needed for intra-vacuolar proliferation of *L. pneumophila* but neither impacts the normal trafficking and biogenesis of the LCV (Al-Khodor et al., 2008; Habyarimana et al., 2008, 2010; Price et al., 2009, 2010a,b, 2011; Lomma et al., 2010). However, since both AnkB and AnkH are needed for intra-vacuolar replication, it is more likely that change in the asparaginyl hydroxylation status of these injected effectors contributes to the intra-vacuolar growth defect in a lysosomal evasion-independent manner. Indeed, we observed that substituting the hydroxylated residues in AnkB significantly impairs its function which correlates to the data obtained using chemical inhibition and RNAi depletion of FIH activity. The lysosomal evasion-dependent mechanism may involve both injected effectors, though no single injected effector to date has been shown to be required for the ability of the LCV to evade the lysosomes (de Felipe et al., 2008; Isberg et al., 2009; Zhu et al., 2011). A number of host proteins involved in vesicular trafficking have been identified on the LCV (Urwyler et al., 2009; Hoffmann et al., 2014; Bruckert and Abu Kwaik, 2015), and a preliminary bioinformatic analysis of the human genome suggests that the FIH hydroxylation motif is found in a number of these including AP1G1, CopB2, ATP6AP1, CLTC, and PICALM (data not shown). It is possible that alterations in the hydroxylation status of these proteins and others may impact trafficking of the LCV, misdirecting it to the lysosome. It is clear that blocking FIH has a broad effect both on injected *L. pneumophila* effectors and host proteins, culminating in a complete block in *L. pneumophila* intra-vacuolar replication.

A central documented role of FIH is in regulation of HIF1, the key transcription factor that modulates expression of numerous genes involved in oxygen homeostasis, metabolism and immune function (Webb et al., 2009). It is possible that the LCV recruits FIH through interaction with Mint3 and MT1-MMP, which are localized to the LCV, analogous to FIH Golgi-localization observed in macrophages (Sakamoto and Seiki, 2009, 2010). In macrophages, membrane-associated FIH is inactive, at least in terms of HIF1 hydroxylation activity, but through its binding to Mint3 it enables HIF1 to promote transcription of glycolytic genes that are needed by the macrophage to generate ATP (Sakamoto and Seiki, 2009, 2010). *L. pneumophila* uses host amino acids as the primary source of carbon and energy by AnkB-dependent proteasomal degradation, but exogenous pyruvate alone can compensate for proteasomal degradation to enable intra-vacuolar replication of *L. pneumophila* (Price et al., 2011). This indicates that host pyruvate is an additional metabolite scavenged by intra-vacuolar *L. pneumophila*. Therefore, a consequence of FIH recruitment to the LCV may be increased HIF1 activity, which will ultimately increase availability of pyruvate that the bacteria can scavenge from the intracellular environment to use as an energy source and building block of macromolecules.

The biological consequence of FIH-dependent asparaginyl hydroxylation of many ARD-containing proteins remains unclear, but is likely to impact function and/or sub-cellular localization of these proteins in the host cell. For *L. pneumophila*, host FIH is essential for intra-vacuolar proliferation, and this involves lysosomal evasion-dependent and -independent mechanisms, which likely involves hydroxylation of both injected effectors and host proteins. Additionally, we identified effectors from other bacterial pathogens that harbor type III and IV secretion systems that potentially undergo asparagine hydroxylation (Table S1), suggesting this post-translational modification is a potential general paradigm in host-pathogen interaction.

## Author contributions

Conceptualization, CP, OSF, and YK; Investigation, CP, MM, SJ, AB, JV, and NA; Resources, ML and OSF; Writing—Review & Editing, CP, MM, SJ, AB, JV, ML, NA, OSF, and YK; Funding Acquisition, YK.

## Funding

The YK lab is supported by Public Health Service Awards R01AI120244 and R21AI116517 from the NIAID and by the Commonwealth of Kentucky Research Challenge Trust Fund. AB was supported by a National Science Foundation Graduate Research Fellowship (NSF GRFP) under Grant No. DGE- 1144204.

### Conflict of interest statement

The authors declare that the research was conducted in the absence of any commercial or financial relationships that could be construed as a potential conflict of interest.
