# Supplementary material for: Host FIH-Mediated Asparaginyl Hydroxylation of Translocated Legionella pneumophila Effectors
Source: Front Cell Infect Microbiol. 2017 Mar 6;7:54. doi: 10.3389/fcimb.2017.00054 (PMC5337513; doi:10.3389/fcimb.2017.00054)
Supplement: Supplementary file 1 [file DataSheet1.pdf]

Host FIH-mediated asparaginyl hydroxylation of translocated *Legionella pneumophila* effectors

Christopher Price[1], Michael Merchant[2], Snake Jones[1], Ashley Best[1], Juanita Von Dwingelo[1], Matthew Lawrenz[1,3] Nawsad Alam[4], Ora Schueler-Furman[4] and Yousef Abu Kwaik[1,3]

[1]Department of Microbiology and Immunology, [2]Department of Medicine-Renal, [3]Center for Predictive Medicine, College of Medicine, University of Louisville, KY, [4]The Hebrew University of Jerusalem Department of Microbiology and Molecular Genetics, Faculty of Medicine, Hadassah Medical School Institute for Medical Research Israel-Canada (IMRIC)

Table S-1: Type III and IV translocated effectors harboring the FIH asparaginyl hydroxylation recognition motif.

Gene	Motif	Cellular location	Function	Intracellular Growth defect
Legionella pneumophila				
LepB	$^{1047-}$LKKKLTELNV^{-1056}	LCV	Rab1 GAP	Defective in amoeba<br>(impaired release)<br>Not defective in macrophage
LegK1	$^{187-}$LSEEVPELNL^{-196} `	Ectopic LegK1-GFP diffuse in cytosol	Ser/Thr kinase<br>Activate NF-κB<br>IκBα phosporylation	No defect
LegC4	$^{331-}$LHWKIKEINL^{-340}	Unknown	Unknown	Unknown
SdeC	$^{1164-}$LNNIQQELNL^{-1173}	LCV	SidE paralog<br>Function unknown	Defective in amoeba<br>Not defective in macrophage
SdeB	$^{1169-}$LNNIQQELNL^{-1178}	(Likely LCV?)	SidE paralog<br>Function unknown	No defect
SdcA	$^{654-}$LQRVSQEINA^{-663}	LCV	SidC paralog<br>Ubiquitin ligase	No defect
LubX	$^{22-}$LREAALEANL^{-31}	Unknown	E3 ubiquitin ligase targeting host Clk1 and SidH effector	No defect in macrophage<br>Mutant exhibits increased killing of Drosophila
SidH	$^{991-}$LKDIHSELNA^{-1000}	Unknown	Stability regulated by LubX	No defect (Drosophila only tested)
AnkB	$^{113-}$LWRKYTFDPNF^{-123}	LCV	F-box protein<br>Recruit polyUb to the LCV<br>Generation of amino acids for replication	Defective in amoeba<br>Defective in mammalian cells<br>Defective in mouse model
AnkH	$^{51-}$LIARKVDINK^{-60}<br>$^{85-}$LTYGADPNA^{-93}	Punctate distribution	Unknown	Defective in amoeba<br>Defective in mammalian cells<br>Defective in mouse model
AnkN	$^{758-}$LLKEGVDLNK^{-767}	Punctate distribution	Phosphocholination of Rab1	No defect
Yersina pestis				
YopM	$^{315-}$LPPSLEELNV^{-324}		Block inflammasome activation	
Shigella flexneri				
IpaH4.5	$^{80-}$LPLHIRELNI^{-89}		E3 ubiquitin ligase	
Rickettsia felis				
Ank	$^{166-}$LLKYGADINA^{-175}	Unknown	Unknown	Unknown

Table S-2: Relative gene expression levels of HIF1-dependent genes in hMDMs treated with hydroxylase inhibitors NODA and DMOG

Gene	DMOG	NODA
GLUT1	7.6	16.7
GLUT3	13.5	10.6
LDHA	3.8	3.3
PGK1	4.4	3.8

* All fold increases are statistically significant (p-value <0.001) using REST-XL software

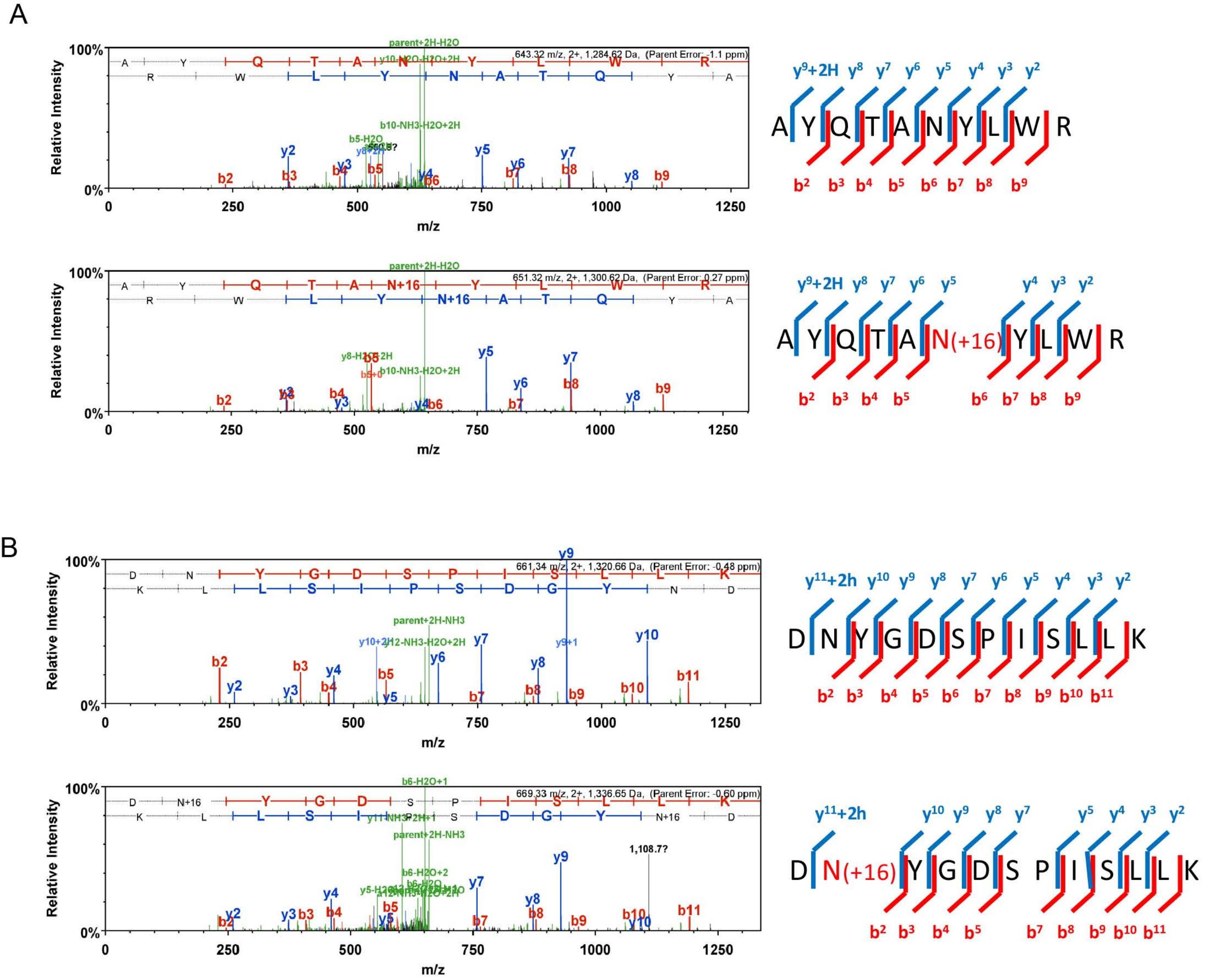

Fig S1. AnkB is modified by host asparaginyl hydroxylation. High resolution LCMS analysis 1D-LC-LTQ-Orbitrap-ELITE-MS) of AnkB protein expressed in HEK293T cells identifies hydroxylated asparagine residues. A) AnkB: (Top) CID fragmentation spectrum for +2 charged ion with a monoisotopic m/z: 1,284.62 Da; (Bottom) CID fragmentation spectrum for +2 charged ion with a monoisotopic m/z: 1,300.62 Da; ProteomeDiscover v1.4 analysis of upper MS/MS data set identifies a tryptic peptide (Mascot Ion Score 57.2) with the sequence AYQTANYWR. Analysis of lower MS/MS data set identifies a tryptic peptide (Mascot Ion Score 38.4) with the sequence AYQTAN(OH)YWR. Both peptides were observed with <2ppm mass accuracy and with near complete b-ion (red hash) and y-ion (blue hash) coverage of parent ions. B) AnkB: (Top) CID fragmentation spectrum for +2 charged ion with a monoisotopic m/z: 1,320.66 Da; (Bottom) CID fragmentation spectrum for +2 charged ion with a monoisotopic m/z: 1,336.65 Da; ProteomeDiscover v1.4 analysis of upper MS/MS data set identifies a tryptic peptide (Mascot Ion Score 72.6) with the sequence DNYGDSPISLLK. Analysis of lower MS/MS data set identifies a tryptic peptide (Mascot Ion Score 41.6) with the sequence DN(OH)YGDSPISLLK. Both peptides were observed with <1ppm mass accuracy and with near complete b-ion (red hash) and y-ion (blue hash) coverage of parent ions.

A

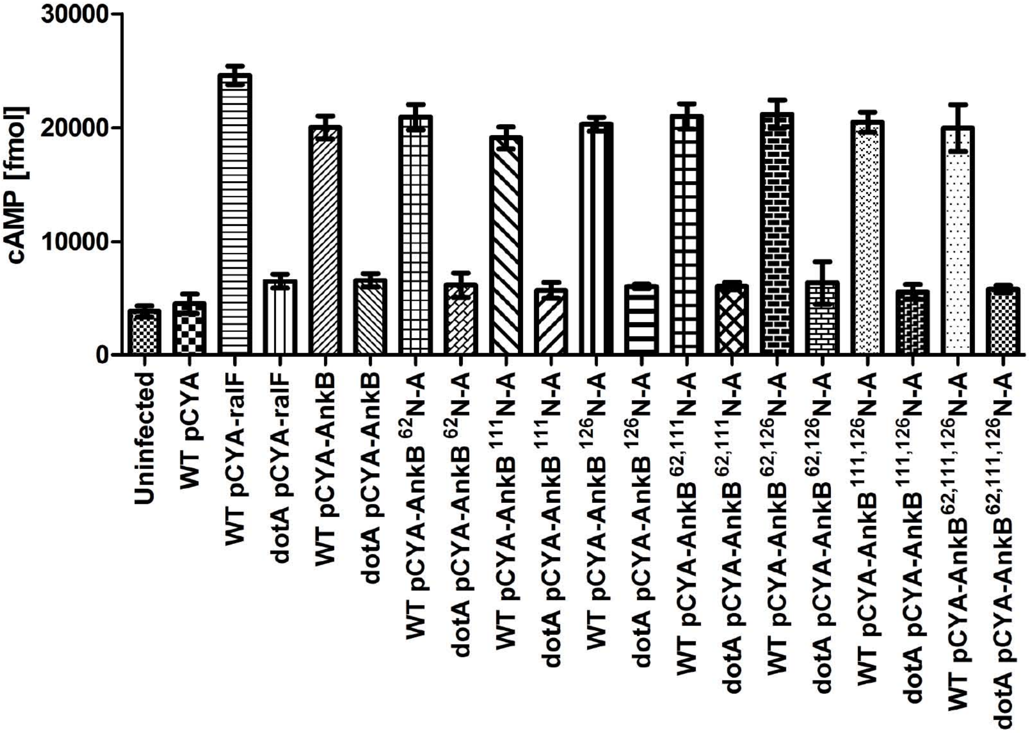

B

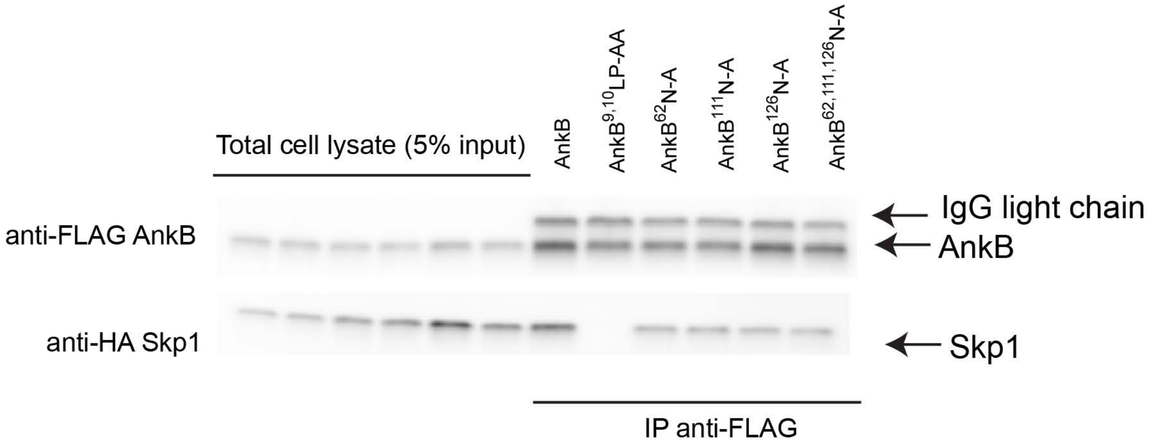

Fig S2: Substitution of hydroxylated asparagine residues in AnkB does not impact translocation into host cells or interaction of this effector with Skp1. A) Adenylate cyclase translocation assay of AnkB and various asparagines substitution mutants in wildtype and dotA mutant L. pneumophila. hMDMs were infected with bacteria at an MOI of 20 for 1h in triplicate and cAMP production assessed by ELISA. B) Co-immunoprecipitation of AnkB and Skp1 in co-transfected HEK293T cells is not impacted by substitution of hydroxylated asparagine residues. The band above AnkB in the anti-FLAG western blot represents the IgG light chain. Following anti-FLAG western analysis, the blot was stripped and re-probed with anti-HA antibody to detect HA-tagged Skp1.

Fig S2

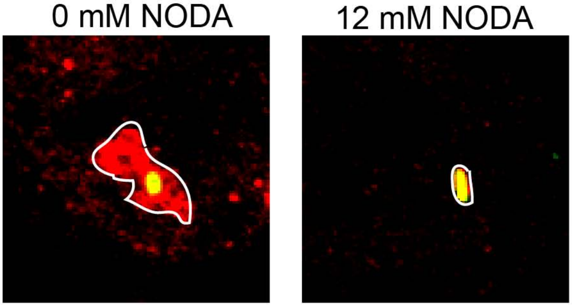

Fig S3: Assessment of ‘ubiquitin’ cloud size surrounding LCVs in infected hMDMs. Z-stack images were generated by confocal microscopy and then analyzed using Olympus FV-10 3.1 software to calculate area within the encircled region.
